# Identification of Direct and Indirect Social Network Effects in the Pathophysiology of Insulin Resistance in Obese Human Subjects

**DOI:** 10.1371/journal.pone.0093860

**Published:** 2014-04-07

**Authors:** Christian H. C. A. Henning, Nana Zarnekow, Johannes Hedtrich, Sascha Stark, Kathrin Türk, Matthias Laudes

**Affiliations:** 1 Institute of Agricultural Economics, University of Kiel, Kiel, Germany; 2 Department of Internal Medicine 1, University of Kiel, Kiel, Germany; University of Bremen, Germany

## Abstract

**Objective:**

The aim of the present study was to examine to what extent different social network mechanisms are involved in the pathogenesis of obesity and insulin-resistance.

**Design:**

We used nonparametric and parametric regression models to analyse whether individual BMI and HOMA-IR are determined by social network characteristics.

**Subjects and Methods:**

A total of 677 probands (EGO) and 3033 social network partners (ALTER) were included in the study. Data gathered from the probands include anthropometric measures, HOMA-IR index, health attitudes, behavioural and socio-economic variables and social network data.

**Results:**

We found significant treatment effects for ALTERs frequent dieting (p<0.001) and ALTERs health oriented nutritional attitudes (p<0.001) on EGO's BMI, establishing a significant indirect network effect also on EGO's insulin resistance. Most importantly, we also found significant direct social network effects on EGO's insulin resistance, evidenced by an effect of ALTERs frequent dieting (p = 0.033) and ALTERs sport activities (p = 0.041) to decrease EGO's HOMA-IR index independently of EGO's BMI.

**Conclusions:**

Social network phenomena appear not only to be relevant for the spread of obesity, but also for the spread of insulin resistance as the basis for type 2 diabetes. Attitudes and behaviour of peer groups influence EGO's health status not only via social mechanisms, but also via socio-biological mechanisms, i.e. higher brain areas might be influenced not only by biological signals from the own organism, but also by behaviour and knowledge from different human individuals. Our approach allows the identification of peer group influence controlling for potential homophily even when using cross-sectional observational data.

## Introduction

Obesity is becoming a major health problem in many countries throughout the world with the increasing prevalence reaching almost epidemic proportions [1]. Of particular concern, obesity associated co-morbidities such as type 2 diabetes and cardiovascular disease are driving a progressive increase in biomedical and also socio-economic problems.

In the past, epidemiological studies revealed a significant correlation of the risk of childhood obesity with parental BMI, suggesting a genetic impact in the development of this important metabolic disease [2]. Subsequently, several studies identified risk alleles for obesity, with most of them being involved in central appetite regulation in distinct brain areas in the hypothalamus. These risk alleles included SNPs in Proopiomelanocortin [3], Neuropeptide-Y [4], Leptin [5], Agouti-related Peptide (AgRP) [6] and, of particular importance, in the Melanocortin-4-receptor (MC4R) [7]. The identification of a central role of specific neurons within the hypothalamus in the pathophysiology of obesity lead to further experimental studies in affected human subjects in order to investigate if also distinct areas in the cerebral cortex are somehow involved in the abnormal regulation of eating behaviour. These studies identified regions in the medial frontal and middle frontal gyrus, which are important in dysregulation of reward activity in the brains of obese human subjects [8]. In contrast to the basal brain functions organised in the hypothalamus, these higher brain areas might be influenced not only by biological signals from the own organism, but also, for example, by behaviour and knowledge from different human individuals [8], [9].

Beyond these intrinsic neurobiological mechanisms, a broad set of social and environmental explanations have been provided for surging rates of obesity [10]. In particular, recent studies support the role of social networks as a determinant of the prevalence of obesity [11], [12], [13], [14], [15] or health outcomes in general [16], [17]. One of the seminal studies showing that social networks are important in the spread of obesity was reported by Christakis and Fowler in 2007 [11]. In this analysis, based on the Framingham Heart Study, it was reported that the risk for becoming obese for a human subject is increased by 57% if he or she had a friend who became obese in a given interval. This finding was particularly interesting, since in the same analysis, the risk of developing obesity was only 40% increased if a sibling became obese. Interpreting their results Christakis and Fowler argue that obesity is “contagious”, transmission being mediated by changing weight–related behaviour (diet, exercise, lifestyle, etc.) [11]. However, the specific mechanisms by which networks influence behaviour are not fully understood, yet, although social norms [11], [16], [18], imitation [11], [19], belief formation as well as social capital have been implicated [20]. Moreover, at the methodological level the social network ‘contagion’ hypothesis has also been critically discussed [12], [21], [22], [23]. Existing empirical studies try to identify network effects using observational data, hence these studies are plagued by serious identification problems, which can be best summarized by Manski's reflection problem [24]. In this regard Shoham et al. [10] applied the Stochastic Actor-Based Model (SABM) as an innovative statistical approach developed by Snijders [25] to distinguish homophily from social contagion (see table 1: glossary). Although we consider the SABM as an appropriate statistical model to solve the identification problem between contagion and homophily, a drawback of this approach, however, can be seen in the fact that it demands longitudinal data, while many clinical studies provide only cross-sectional data.

**Table 1:** Glossary.

Term	Definition
EGO	The actor whose network and behavior choices are being modeled.
ALTER	A person connected to the ego who may influence the behavior of the ego. An actor who is named as a friend by the ego.
Actor	A respondent.
Homophily	The tendency for people to choose relationships with people who have similar attributes.
Peer influence	The effect of alters' behavior on ego's behavior.
Social Influences	Synonym for peer influence.
Tie	A connection between two individuals (nodes) that can be either one-way (directed) or two-way (bilateral)
Node	An object that may or may not be connected to other objects in a network.
EGO-centric network	Subset of social relations among all persons (ALTERS) to whom a specific individual person (EGO) has a social tie. The EGO-centric network is also called the neighborhood or peer group of EGO.
Network multiplier	The value of similar behaviors or attitudes, averaged across all of the EGO's ALTERs; network multiplier is used as a measure of peer influence.
PSM	Propensity Score Matching is a non-parametric econometric estimation method of treatment effects controlling for potential selection bias.
SABM	Stochastic Actor-Based Model.

In this context, the aim of the present study was to develop an innovative approach using cross-sectional observational data (1) to test empirically the effect of social networks on the development of obesity in an independent European cohort and, most importantly, (2) to examine the effect of social networks on insulin resistance in obese human subjects.

## Material and Methods

### Data collection and measurement

We conducted a clinical and social survey [Food Chain Plus Study, funded by the federal ministry of education and research (BMBF), Number: 0315540A, DRKS00005285]. The survey started in September 2011 and has enrolled to date a subsample of 327 obese people with a BMI >30, and a randomized control group of 350 probands. The study was approved by the local ethics committee (Number: 156/03, Ethics committee of the University of Kiel, Germany) and written informed consent was obtained for every subject before inclusion into the study. For each proband we collected anthropometric (weight, height, blood pressure, waist circumference, sensory testing, testing for muscular strength), and biochemical data [fasting insulin serum levels, fasting glucose serum levels, serum C-reactive protein levels, serum triglyceride levels] as well as behavioural data. The biochemical analysis was performed by routine measurements within the department of laboratory medicine at the University Medical Centre in Kiel. The Homeostasis Model Assessment Index for Insulin Resistance (HOMA-IR) was calculated as follows: fasting insulin (μU/ml)*fasting glucose (mg/dl)/405. Probands visited the University Medical Centre in Kiel where relevant anthropometic and biochemical data has been collected. Moreover, diabetes type 2 was diagnosed. In total 28 of the 677 probands had diabetes type 2. Since medication for diabetes type 2 might reduce the HOMA-IR index we explicitly checked the HOMA-IR index of the 28 EGOs with diabetes type 2 (for the term EGO see table 1: glossary). None of the 28 probands had a normal HOMOR-IR index below or equal to 2. A contrario, for most EGOs with diabetes type 2 an extremely high HOMA-IR index is reported resulting in an average HOMA-IR index of 19.1 for this specific subgroup. Accordingly, we concluded that including the 28 EGOs with diabetes type 2 in our sample will not bias our results (We thank an anonymous reviewer for pointing out the potential impact of medication for diabetes type 2 in the HOMA-IR index.).

Further, data on nutrition and activity behaviour as well as relevant socio-economic characteristics of probands were collected. For further details please see table 2. Socio-economic data included age, sex, education, household size, and household income. Behavioural and lifestyle data that were collected for ALTER including frequency of undertaking diets (DIET), attitude towards food (AT), nutritional knowledge (KNOW), frequency of physical activities (SPORT). The data were collected for EGO as well as for all of EGO's social network contacts (ALTER). The collected data is reported in table 2. Moreover, in a special social network survey we collected EGO-centric network data from each proband. Moreover, EGO-centric network data had been collected when probands visited the study center using a specific computer based social network questionnaire. The collected ego-centric network data were applied to the name generator concept, the state-of-the-art methodology to collect social network data [26]. To implement this, the following three name generator questions were asked:

**Table 2:** Summarized descriptive statistics of the FOCUS-sample.

	EGO (Std.deviation)	ALTERS
	*mean values*	*mean values*
Age	51.05	48.89
	(14.46)	(12.85)
Sex (1: male, 0: women)	1: 232, 0: 452	1: 1241 , 0:1759
Household-Size (HS)	2.35	
	(1.62)	
Income^1^	8.92 (4.01)	
Education^2^ (EDUC)	5.32 (2.32)	^3^3.92 (1.84)
BMI	32.88 (10.98)	42.8° (0.71)
HOMA-IR	4.93 (7.15)	
N	684	3033
Behavior		
Knowledge^5^ (KNOW)	4.93 (1.85)	3.64 (0.74)^6^
Attitude^7^ (AT)	4.14 (1.58)	1.75 (0.54)^8^
Diet^9^	4.93 (1.85)	2.30 (1.08)^10^
Sport		2.55 (1.01)^11^
Network		
Size	5.54 (2.93)	
Duration (years)	25.56 (11.33)	
Type (0: family, 1: friends)	0: 1186; 1: 1847	
Frequency^12^	3.11 (0.61)	
Intensity^13^	2.55 (0.41)	
Multiplier		
Knowldge (KNOW)	6.83 (2.39)	
Attitude (AT)	3.34 (1.39)	
Diet	4.04 (2.53)	
Sport	4.66 (2.33)	
BMI	5.07 (2.09)	

1 Income level: 1: <499 Euro to 16: >4000 Euro.

2 Education: 1 =  means no educational achievement; 10 =  PhD.

3 Education: 1 =  means no educational achievement; 8 =  PhD.

4 Nutritional status: 1 =  very slim; 5 =  very fat.

5 Reading of food information is important: 1 (not agree)-7 (agree completely).

6 Knowledge: 1 =  no; 5 =  excellent.

7 Low fat food is important: 1 (not agree)-7 (agree completely).

8 Attitude: 1 =  food has to be tasty, 2  =  food has to balance enjoyment of eating and health, 3 =  food has to be mainly healthy.

9 I always eat healthy and well balanced: 1 (not agree)-7 (agree completely)

10 Diet: 1 =  never; 5 =  more than 5 times.

11 Sport: 1 =  never; 5 =  daily.

12 Contact frequency: 1 =  never to 5 =  daily.

13 Intensity: 1 =  no talk about private issues; 3 =  often.

*G1: With whom do you regularly discuss personal problems?*

*G2: To whom can you turn for help if you have a problem?*

*G3: With whom do you regularly discuss health-related (especially weight-related) problems?*

For all ALTER mentioned by EGO in response, we also asked for their *gender, age, education*, and *profession*. Further, we asked EGO to estimate for each ALTER the following characteristics: (1) ALTER-*BMI* measured in five categories (1–5) ranging from very slim to very fat, for details see table 2; (2) *Nutrition knowledge (ALTER-KNOW)*: 1 = very low, 2 = low, 3 = average, 4 = good, 5 = excellent; (3) *Nutritional attitude (ALTER-AT)*: 1 =  food is mainly convenience; diet has to balance health and convenience aspects, 3 = diet has to be mainly healthy; (4) *Frequency of sport activities* (ALTER-SPORT) longer than 30 minutes: 1 = never; 2 = 1–2 per month, 3 = 1 per week, 4 = several times per week; 5 =  every day; (5) *Diet behaviour (ALTER-DIET)*, we ask how often ALTER has made a specific diet to lose weight: 1 = never, 2 = 1 time, 3 = 2–3 times; 4 = 4–5 times, 5 =  >5 times. At the end of the questionnaire, we also asked questions about the *strength, length*, and *importance* of the relation with the named individuals. Following the concept of Krackhardt (for further explanations see [26]), we asked interviewees to describe the pairwise relations of the ten most important individuals mentioned on a 3 point scale with 0 =  do not know each other, 1 =  know each other, 2 =  know each other very well.

### Data management and statistical analysis

We calculated different network multiplier (NET-Z) measuring the field strength of different health-relevant behaviours and attitudes (Z =  KNOW, DIET, BMI, AT, SPORT) prevalent in EGO's social network and operating on EGO [27]:
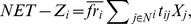
where 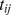 is the relative strength of a network tie between EGO 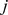 and ALTER 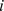 and 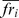 is the average absolute strength of a network tie. We measure the relative strength of EGO-ALTER relations 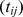 using the relation frequency of ALTER's network contact with EGO. Our network multiplier also corresponds to the *network force*, a measure suggested by [28] as well as to the position generator, a EGO-centric network measure suggested by [28], [29].

First, we apply Propensity Score Matching (PSM) to identify the average treatment effect on treated (ATT) [30] of the different lifestyle attributes of EGO's social peer group (NET-Z).

To this end we define for each attribute NET-Z a binary treatment variable 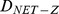 as follows:
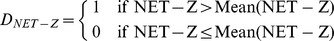



Further, we estimated separate probit functions using each D_NET-Z_ as endogenous variable and relevant socio-economic characteristics 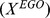 and lifestyle indicators of EGO 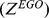 as well as all relevant lifestyle multiplicators calculated for EGO's social network except the one corresponding to the endogenous variable D_NET-Z_. In particular, socio-economic variables include EGO's age, sex, household size (HS), education (EDUC), while EGO's lifestyle indicators include EGO-attitude towards food (EGO-AT), EGO's nutrition knowledge (EGO-KNOW), EGO's diet behaviour (EGO-DIET). Based on each estimated probit function we calculated corresponding PSM-scores for all EGO's and used calculated PSM-scores to match the treatment group 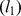 with a corresponding control group 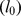 applying a Kernel Matching operator [31], [32]. Finally, we calculated for each treatment variable NET-Z the average treatment effect on treated as follows:
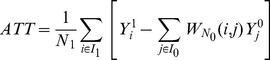



Using the Kernel matching operator implies that all members of the control group are used to estimate the ATT, but with different weights, where the following weight for each observation in the control group is used:
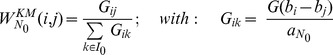



G denotes the Kernel function [31] with 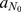 and 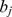 being specific parameters of the Kernel function. The kernel weights decrease with the distance of the propensity score of a member of the control group to the propensity score of the member of the treatment group. Thus, treated EGOs are compared with non-treated EGOs who have the same socio-economic characteristics as well as the same health related attitudes and behavior as treated EGO and who also have the same peer group characteristics despite from the treatment variable. PSM-matching is a statistical procedure that allows the construction of a control group including other EGOs that are statistical siblings of the treated EGOs, but differ exactly regarding the considered treatment variable. Since, EGO's own characteristics are explicitly included in the statistical construction of the control group PSM matching controls for a potential selection bias due to dynamic peer group selection such as homophily, but also other potential selection biases assuming all relevant determinants of selection into treatment are taken into account (‘selection on observables’ see [31]).

Second, to analyze to what extend social networks have an influence on insulin resistance and thus on EGO's probability to develop type 2 diabetes and cardiovascular diseases, we regress EGO's HOMA-IR-index on EGO's obesity-status, socio-economic variables (X) and health related lifestyle attributes (EGO-Z) as well as on the corresponding social network multipliers (NET-Z) measuring the field strength of relevant lifestyle attributes of EGO's social network operating on EGO. EGO's obesity status is measured by a dummy variable, where OBS-EGO = 1 indicates a BMI>30 and OBS-EGO = 0 indicates a BMI<30. Regression models are estimated applying a two-stage IV-estimator to take potential endogeneity of EGO's BMI into account. We used EGO's lifestyle attributes (EGO-Z), the relevant network multipliers of EGO's social network (NET-Z) and EGO's socio-economic variables (X-EGO) as instruments for EGO's obesity status (OBS-EGO). Moreover, we use the second stage estimation to analyze the indirect impact of social networks on EGO's HOMA-IR-index via influencing EGO's obesity status (OBS-EGO), while the main regression at the first stage includes direct effects of social peer groups on EGO's insulin resistance, i.e. direct peer group effects correspond to effects operating via the direct influence of higher brain areas by the behaviour of other human individuals [8] that do not operate via a change in EGO's behaviour or obesity status.

However, since an average of 39% of EGO's network contacts are family ties, estimations might be plagued by an endogeneity problem in the following sense. Insulin resistance is at least partly genetically determined [33]. Hence, assuming that health related behaviour and attitudes are also at least partly genetically determined might imply a spurious relationship between direct peer group effects induced by family ties and EGO's HOMA-index. Of course, we already control for this spurious relationship as well as for a potential spurious correlation due to homophily as a dynamic peer group selection since we explicitly include EGO's own health related behaviour and attitudes in our main regression equation. Nevertheless as an additional robustness check we undertake a three stage IV estimation where we instrumented EGO's family peer group behaviour and attitudes at a third stage using corresponding behaviour and attitude of EGO's non-family ties as instruments. Moreover, we re-estimate our two-stage IV regression model excluding family ties completely.

## Results

### Characteristics of the study cohort

N = 677 subjects designated as EGOs were included. Basic descriptive statistics of our sample are reported in table 2. Any person to whom EGOs are linked serve as a social contact, and is designated “ALTER” in the following. A total of n = 3033 ALTERS, observed family and social ties, were connected. This yields an average of 5.5 ties per EGO within the network. A total of 39.1% of the 3033 ALTERs were family contacts. The remaining 60.9% were connected through friendship to EGO. The average duration of relationship was 25.5 years with a standard deviation of 11.3. The minimum duration was 0.666 years and the maximum 61.5 years. The mean age of investigated EGOs was 51 years with a range from 19 to 84 years. The mean age of ALTERs was 48 years, ranging from 11 to 91. 34% of the EGOs were men, while 41% of ALTERs were male. The educational level was measured on a scale ranging from 1 to 10 (1 to 8 in case of ALTERs) with 1 indicating no formal education and a 10 (8) denoting a PhD-level. The average educational level of EGO's was 5.3 on a 10 point scale, while the mean education level of ALTERs was 4.1 on a 8 point scale. The frequency of EGO's network contacts ranged from 28% who meet daily, over 39% who meet weekly to 29% who meet only on a monthly basis. Only 4% of ALTERs did meet less than one time per month by EGO.

### Treatment effects of social network characteristics on obesity

In table 3 the treatment effects (ATT) of social networks on EGO's BMI are reported, generated from PSM-Matching analysis for the five different network characteristics. As can be seen from table 3 the PSM-matching results imply that the average BMI of EGO's social peer group has no significant influence on EGO's own BMI, while we found significant network effects for the weight related behaviour and attitude of social peer groups. In particular, we found significant treatment effects for diet behaviour and nutritional attitude, while nutritional knowledge and sport activities of peer groups had no significant impact on EGO's BMI. In quantitative terms the impact was the highest for diet behaviour resulting in an average treatment effect of 4.3, i.e. having a social network that frequently engaged in diets implies an increase in BMI by 4.3 kg/m^2^. Given an average BMI of 31 in our sample this corresponds to a remarkable effect of more than 13%, which is highly significant with a t-value of 4.3 (p<0.001). Analogously, we found a remarkably high effect on EGO's BMI of 3.5 kg/m^2^ for health oriented attitude of EGO's social network corresponding to a reduction of over 10% of the average BMI. Furthermore, a high frequency of sport activities in EGO's social network reduces EGO's BMI by 0.75 kg/m^2^, but this effect was statistically not significant.

**Table 3:** Estimated treatment effects (ATT) of social network characteristics on obesity (BMI).

Treatment Variable	Selection	Treated Group	Control Group	ATT	t-values*	p
D_NET-DIET_	unmatched	35.900	31.050	4.849	5.7	0.000
	matched	35.900	31.613	4.287	4.3	0.000
D_NET-BMI_	unmatched	33.357	32.543	0.814	0.96	0.567
	matched	33.357	33.203	0.154	0.14	0.779
D_NET-Sport_	unmatched	31.830	33.652	−1.822	−2.13	0.005
	matched	31.830	32.582	−0.752	−0.71	0.200
D_NET-Know_	unmatched	32.939	32.871	0.068	0.08	0.900
	matched	32.939	34.342	−1.403	−0.8	0.200
D_NET-AT_	unmatched	32.210	33.520	−1.310	−1.54	0.050
	matched	32.210	35.753	−3.543	−2.95	0.000

*t-values derived via bootstrapping.

### Multiple regression analysis on social network effect on insulin resistance

Results of the second stage of the IV-estimations are reported in table 4 (Model A), while results of the probit estimation of the first stage of our IV-estimation are reported in table 5 (Model A). As can be seen from table 4 the main determinant of EGO's insulin resistance corresponds to EGO's obesity status with a normalized partial impact of *0.258*. In absolute terms an increase of 1% in the probability of becoming obese implies an increase of *0.07* units of the HOMA-IR-index. However, beyond obesity status also sex has a significant effect on insulin resistance with a normalized coefficient of 0.101 (see table 4, model A). In particular, males generally have a higher HOMA-IR-index when compared to females, with an absolute difference of 1.44 between men and women.

**Table 4:** Results of the IV-estimation: Dependent variable EGO's HOMA-IR.

	Model A-0			Model A			Model B		
	Coef.	P>|t|*	Beta	Coef.	P>|t|*	Beta	Coef.	P>|t|*	Beta
Male	1.388	0.009	0.098	1.439	0.007	0.101	1.459	0.005	0.103
EGO-DIET	0.140	0.436	0.038	0.140	0.433	0.038	0.184	0.045	0.051
EGO-AT	0.165	0.413	0.039	0.154	0.383	0.036	0.109	0.119	0.026
EGO-KNOW	−0.067	0.630	−0.020						
NET-AT	−0.051	0.840	−0.011						
NET-DIET	−0.291	0.040	−0.110	−0.267	0.053	−0.101	−0.082	0.671	−0.031
NET-BMI	0.284	0.060	0.088	0.332	0.014	0.103	0.245	0.022	0.076
NET-KNOW	0.126	0.465	0.045						
NET-SPORT	−0.264	0.040	−0.092	−0.225	0.038	−0.078	−0.202	0.015	−0.070
EGO-BMI	7.375	0.000	0.263	7.231	0.000	0.258	6.358	0.000	0.227
Prob >F =	0			0			0		
R-squared	0.386			0.385			0.374		
Adj R-squared	0.377			0.379			0.367		

*t-values derived via bootstrapping.

**Table 5:** Results of Logit-Model: Dependent variable EGO's BMI-status (OBS-EGO) (IV-first stage).

	Model A			Model B		
OBS-EGO	coef	P>|z|	Marginal effect	coef	P>|z|	Marginal effect
NET-KNOW	0.031	0.613	0.008	−0.044	0.725	−0.011
NET-AT	−0.134	0.145	−0.033	−0.186	0.340	−0.046
NET-BMI	−0.034	0.536	−0.009	−0.232	0.048	−0.057
NET-DIET	0.238	0.000	0.059	0.504	0.000	0.124
NET-SPORT	−0.001	0.988	0.000	0.026	0.783	0.006
Age	−0.011	0.110	−0.003	−0.014	0.039	−0.004
Male	−0.053	0.789	−0.013	0.009	0.965	0.002
Income	−0.053	0.041	−0.013	−0.066	0.012	−0.016
Education	−0.268	0.000	−0.067	−0.265	0.000	−0.065
HS	0.093	0.252	0.023	0.069	0.333	0.017
EGO-DIET	0.294	0.000	0.073	0.280	0.000	0.069
EGO-AT	−0.384	0.000	−0.096	−0.378	0.000	−0.093
EGO-KNOW	0.144	0.002	0.036	0.149	0.002	0.037
Constant	1.170	0.030		1.883	0.014	

The most remarkable finding of our analyses, however, was a significant and robust direct influence of social networks on EGO's insulin resistance, even correcting for EGO's BMI. In particular, a higher frequency of diet behaviour (p = 0.052) as well as sport activities (p = 0.038) in EGO's social network reduces significantly her/his HOMA-IR-index given normalized regression coefficient of −0.101 and −0.078, respectively. In quantitative terms a maximal difference in the diet behaviour of EGO's network changing from no diet to an average of more than 5 diets undertaken per network contact implies an absolute decrease of *1.16* units of the HOMA-IR-index. Taking the normal HOMA-IR-value of <2.0 as a reference this corresponds to a remarkable change of 58%, and even if we compare this with the average HOMA-IR-index in our sample of 4.8 it still corresponds to a remarkable change of 24%. Analogously, a maximal change of the frequency of sport activities in EGO's network from no activities to an average sport activity of at least once per day decreases EGO's HOMA-IR-index *by 1.06* units, which still corresponds to remarkable 53% and 22% compared to the critical HOMA-IR-value of <2.0 and the average HOMA-IR-value of *4.8* in our sample, respectively.

Interestingly, in contrast to the lifestyle of EGO's social peer group EGO's own lifestyle indicators have no significant direct impact on EGO's HOMA-IR-index. However, EGO's lifestyle significantly influences EGO's BMI-status (see table 5, model A), where especially EGO's health oriented nutritional attitude (EGO-AT) reduces significantly EGO's probability to become obese (p = 0.000) with a marginal effect of −0.096 (see table 5, model A). Moreover, a high education (p = 0.000) and income level (p = 0.041) reduce EGO's probability to become obese. Peer group effects on EGO's BMI, however, are less pronounced in the multiple regression analysis. Only for diet behaviour of EGO's peer network a highly significant positive effect on EGO's BMI-status was observed (p = 0.000, see table 5). For all other network multipliers only an insignificant effect resulted from our multiple regression analysis.

Combining the strong and significant impact of the EGO's BMI on her/his HOMA-IR-index estimated at the second stage with the estimation results of the logit regression at the first stage implies a strong indirect social network effect on insulin resistance. We calculated the indirect network effect as the marginal effect of a network multiplier on EGO's probability of becoming obese multiplied by the marginal effect of EGO's obesity status on her HOMA-IR-index. Significant indirect and direct peer group effects were identified for diet behaviour, while sport behaviour and BMI-status of EGO's peer network impact only directly on EGO's insulin resistance (Model A, table 4, 5). In contrast, EGO's own health related lifestyle and nutritional knowledge impact only indirectly on his/her insulin resistance (Model A, table 4, 5). Please note that we essentially derived the same results, i.e. we observe significant direct and indirect peer group effects on EGO's HOMA-IR-index, undertaking a three-stage IV estimation instrumenting behaviour and attitude of EGO's family ties (Model B, table 4, 5). Furthermore, results of our preferred model A do not change if we include insignificant lifestyle variables of EGO and his/her peer network (see model A-0 in table 4). Moreover, these results remain also robust if we re-estimate the two-stage IV regression model excluding family ties (estimation results are not presented here, but are available from the authors upon request). Therefore, we are confident that our main estimation results correspond to robust findings.

## Discussion

In recent years biomedical research has been enormously extended in regard to the pathophysiology of obesity and its associated co-morbidities. In terms of body weight regulation, it was established early that genetic factors explain 30–50% of the obesity epidemic and that environmental factors are tremendously important [34]. While initial studies focused mainly on nutrition, eating behaviour and physical activity as important environmental factors, in 2007 Christakis and Fowler demonstrated that social network effects might even be more important than genetic polymorphisms in the development of obesity [11]. However, in contrast to what was found for obesity, until now no data exist on the impact of social network effects on insulin resistance, a key obesity-associated morbidity that is the mediator of obesity-associated type 2 diabetes, lipid disorders and atherosclerosis [35]. Therefore the aim of the present study was to (1) investigate the impact of social network effects on obesity development in an independent European cohort and (2) to examine potential direct and indirect social network effects on the development of insulin resistance.

The results reported here imply that specific lifestyles attributes of social peer groups, especially frequent diet and nutritional attitude in favour of healthy food, influence significantly EGO's BMI. These findings confirm the previously identified network effects on body weight gain in the US population [11]. Together, these findings provide evidence for the hypothesis that appetite regulation organised in the hypothalamus might be influenced not only by biological signals from the periphery (e. g. ghrelin and leptin [36]), but also via function of the cerebral cortex via knowledge and/or behaviour from different human individuals of the patients relatives and peer group [8], [11].

In addition to what was examined in earlier reports, in the present social network study we also calculated the HOMA-IR index for the first time for each of the 677 individual subjects in order to obtain a measure for their insulin action. Applying our mathematical model, we identified for the first time that social networks can influence EGO's insulin resistance. This is in part explained by indirect effects of the peer group on EGO's BMI and the BMI determining EGO's insulin resistance. This finding is not unexpected since many clinical and experimental studies have shown insulin resistance of liver and skeletal muscle to be associated with obesity [37]. Ectopic lipid accumulation in liver and skeletal muscle in response to an excess of energy intake is postulated to explain this association, leading in turn to serine phosphorylation of insulin receptor substrate (IRS)-1 and thereby inhibition of intracellular insulin receptor signalling [35], [38]. Therefore, as shown in this report, if the peer group influences EGO's BMI then one would expect that EGO's insulin resistance should also be affected. Hence, from a mathematical point of view, the fact that peer group effects on EGO's BMI are in line with peer group effects on EGO's insulin resistance indicates, that the associations found in our cohort are true sociobiological effects rather than statistical artefacts.

The most remarkable finding of our study corresponds to the fact that we also have been able to identify direct peer group effects on EGO's insulin resistance. That means that our regression analyses yield these significant network effects even when we control for EGO's BMI and for EGO's own weight-related attitudes and behaviour, respectively. This is particular interesting, since it has been shown that for example physical activity is able to improve insulin sensitivity in overweight subjects independently of significant changes in BMI [39]. Therefore, the fact that the degree of physical activity of the peer group beneficially affects directly EGO's insulin resistance suggests the existence of a potent sociobiological mechanism in the pathogenesis of insulin resistance.

At a methodological level PSM-matching as well as two-stage multiple regression analysis are adequate methods avoiding the problem of latent homophily even if only cross-sectional data can be used (no panel data). However these statistical analyses are based on certain assumptions that we could not test explicitly. In particular, PSM matching is based on the assumption of “selection on observables” [31], i.e. we have to assume that our analysis includes all relevant selection variables. Thus, to the extent that “selection on unobservables” occurs PSM would deliver biased results [31]. In contrast, our two-stage estimation is not plagued by the problem of latent homophily as it appears rather unrealistic to assume that peer group selection occurs on the basis of insulin resistance. However, since an average of 39% of EGO's network contacts are family ties estimations might be plagued by an endogeneity problem resulting from spurious relationship between direct peer group effects and EGO's HOMA-index induced by genetic relations among family ties and EGO. Given our estimation design this spurious relationship could only occur if weight related behaviour and attitudes are determined by the same genes as the HOMA-index which we consider as rather unrealistic. Nevertheless we undertook an additional robustness check, i.e. we conducted a three stage IV estimation where we instrumented EGO's family peer group behaviour and attitudes at a third stage using corresponding behaviour and attitude of EGO's non-family ties as instruments. Moreover, we re-estimated our two-stage IV regression excluding family ties. Both alternative estimation strategies delivered in essence the same results. Hence, beyond theoretical considerations also on pure statistical grounds we are confident that we can exclude spurious relationships and that our main results correspond to robust findings.

In summary our study indicates for the first time that social network phenomena appear not only to be relevant for the spread of obesity, but also for the spread of insulin resistance. Direct and indirect social network mechanisms have been identified as significant factors determining the risk for impaired insulin signalling. Weight-related attitudes and behaviour of peer groups exert particularly significant impact not only on EGO's obesity status, but also directly on EGO's insulin resistance. These results might have important clinical implications for the design of future obesity therapy programs. Given the fact that many individual-level intervention strategies to prevent obesity, including nutritional education, behavioural therapy and physical activity [40], [41], achieve very few sustained effects [41] our results might be used to design novel weight loss programs. These programs should include not only patients (EGOs) treatment but also education of the patients peer group to achieve more sustained results of multimodal obesity therapy programs in the future. Moreover, beyond designing innovative obesity therapy programs including peer group effects based on external peer group compositions, understanding the dynamics of peer group formation might also enable the design of peer group structures that amplify identified positive peer group effects on EGO's obesity and related co-morbidities.
